# Simultaneous Determination of Residue from 58 Pesticides in the Wheat Flour Consumed in Tehran, Iran by GC/MS

**Published:** 2017

**Authors:** Mohammad Rezaei, Nabi Shariatifar, Shahram Shoeibi, Maryam Amir Ahmadi, Gholamreza Jahed Khaniki

**Affiliations:** a *Department of Environmental Health Engineering, School of Public* *Health, Tehran University of Medical Sciences, Tehran, Iran. *; b *Food and Drug Laboratory Research Center, Food and Drug Organization, MOH & ME, Tehran, Iran. *; c *Food and Drug Reference Control Laboratories Center, Food and Drug Organization, MOH & ME, Tehran, Iran.*

**Keywords:** Pesticides, GC/MS, Wheat flour, Food safety.

## Abstract

Food safety has a direct impact on human health and as such is a growing concern worldwide. Presence of harmful pesticide residue in food is a serious cause for concern among consumers so it is important to monitor levels of pesticides in foods. The aim of this study was simultaneous determination of concentrations of 58 pesticides in 40 wheat flour samples collected from Tehran market in January, 2014. The city under study (Tehran) was divided into five districts and samples were collected independently from each district and sourced from different bakeries (n=40). A gas chromatography-mass spectrometry single quadrupole selected ion monitoring «GC/MS-SQ-SIM» method was used to quantify residue of 58 pesticides in the wheat flour samples. Four of the 40 samples showed contamination with Malathion (2 samples: 50.96 ± 11.38 and 62.088 ± 11.38 ppb) and 2, 4-DDE (2 samples: 19.88±15.24 and 13.7 ± 15.24 ppb). that had levels below MRLs of these pesticides in Iran. Averages of recovery of pesticides at 6 concentration levels were in the range of 81.61-118.41%. The method was proven as repeatable with RSDr in the range of 6.5-29.45% for all concentration levels. The limit of quantification for 37 of the tested pesticides was calculated as 15 ppb and for the other 21 tested pesticides, the concentration was 25 ppb. In summary, results of these tests suggested that the wheat flour consumed in Tehran, was within safety limits in terms of levels of pesticide residues.

## Introduction

Food provides essential supplies of life sustaining nutrients, vitamins, and minerals but it may also contain a variety of naturally-occurring toxins in varying quantities. Some toxins present a health risk to consumers but this usually occurs only when certain food items have not been properly prepared. Research has identified a beneficial role of some of these naturally occurring substances, such as preventing illnesses. However, there is a more important risk of contamination by synthetic compounds such as pesticides. Public policy should be directed toward reducing any unnecessary overburden of pesticide residue in the human diet (1). Cereal production, among other food types, has a historical association to human development. Cultivation of cereal crops has played a very important role in the so-called ‘‘Neolithic revolution’’, in which humans first achieved full control over their food supply. This is accepted as the point that human development shifted to formation of agricultural societies and, with that a new concept of civilization was borne that has partly survived until today (2). Cereals constitute one of the most intensely produced and consumed food products in the world. They are an important global product and an important part of the human diet, as a source of energy and high contents of essential fatty acids, nutritious proteins, and dietary fiber; cereals also supply important minerals, vitamins, and other micronutrients that are essential for the maintenance of optimal health. In particular, cereal grains are the basis of bread, which is one mankind’s earliest food products and that currently constitutes a major food technology. There is currently huge demand for cereals, and cereal derivatives to feed the ever-growing global population and significant effort is needed to improve production yields. Pest control makes an important contribution to maximizing yields. There are more than 1100 pesticides currently registered on the status list of all active pesticide substances in the European Union (EU) market (3). However there is a negative effect from application of pesticides in agricultural practice, many pesticides are harmful to the environment and are known as or suspected to be toxic to humans. There is increasing public concern about the possible health risks of pesticide residue in the diet that has deeply modified strategies for crop protection, with the main emphasis on food quality and safety. Widespread health concerns have led to strict regulation of maximum residue limits (MRLs) of pesticide residue in food products. To date, more than 17,000 European Community MRLs have been set for various commodities for 133 active pesticide substances (4). Pesticides have been used for many years. In earlier times they were used to protect against fungi and insect pests and/or to provide quality preservation (5). This great increase in the use of pesticides has occurred with the development of new organic chemicals following the two major world wars; WWI and WW2. In addition to the chemicals used to control fungi and insects, new developments have been introduced such as; nematocides, herbicides, rodenticides, avicides, defoliants, and wood preservatives (6). Discussions on risks associated with human exposure to pesticides pay particular attention to direct poisoning and fatality, potential induction of cancer, and effects on reproduction and immune and nervous systems, diabetes, neurodegenerative disorders such as Parkinson’s disease, chronic diseases, and genetic damage, epigenetic modifications, endocrine disruption, mitochondrial dysfunction, oxidative stress, endoplasmic reticulum stress, and diminished intelligence (1, 5, 7, 8). In agriculture for wheat production, pesticides such pirimicarb, chlorpyrifos, carbaryl, malathion, propiconazole, tebuconazole and triadimenol are used to prevent infestations of insects, fungi, and weeds. The European Union (EU) has set maximum residue limits (MRLs) for the above-mentioned pesticides in wheat crops at 0.5, 0.05, 0.5, 8, 0.05, 0.2, and 0.2 mg/kg respectively (9).

Today, the most frequently employed analytical approach to determine levels of pesticide in foods is based on mass spectrometry, such as Gas chromatography–mass spectrometry (GC–MS). In the case of Green aspects and environmental risks, Anastassiades *et al*. suggested a simple, safe, cheap, high sample throughput method namely QuEChERS in pesticides residue analysis (10). This study was performed according to the safe QUECHERS method.Over the past decades, approaches to trace level determination of pesticides have moved away from the use of GC with selective detectors including electron capture detection (ECD) (11, 12) and nitrogen phosphorus detection (NPD) (13). To mass spectrometer (MS). detectors which are more sensitive and selective (14). The use of mass spectrometry, with its information-rich content and explicit confirmation, is recommended for monitoring pesticide residues in the entire world (15-17). 

This is the first attempt in Iran using GC-MS-SIM technique and spike calibration curve for simultaneous determination of determine 58 pesticides with difference in physicochemical properties in wheat flour marketed in Tehran, Iran. The other outcome of this study is calculating the estimated daily intake (EDT) of the pesticides via wheat flour and its comparison with the acceptable daily intake (ADI). Methods

*Samples Collection*

Tehran city was divided into five districts and samples from each district was collected independently and equally of different Bakery in January of 2014 (Barbari (n=10), Sangak (n=10). Baguette (n=10) and Lavash (n=10)).

*Sample preparation*

**Figure 1:**
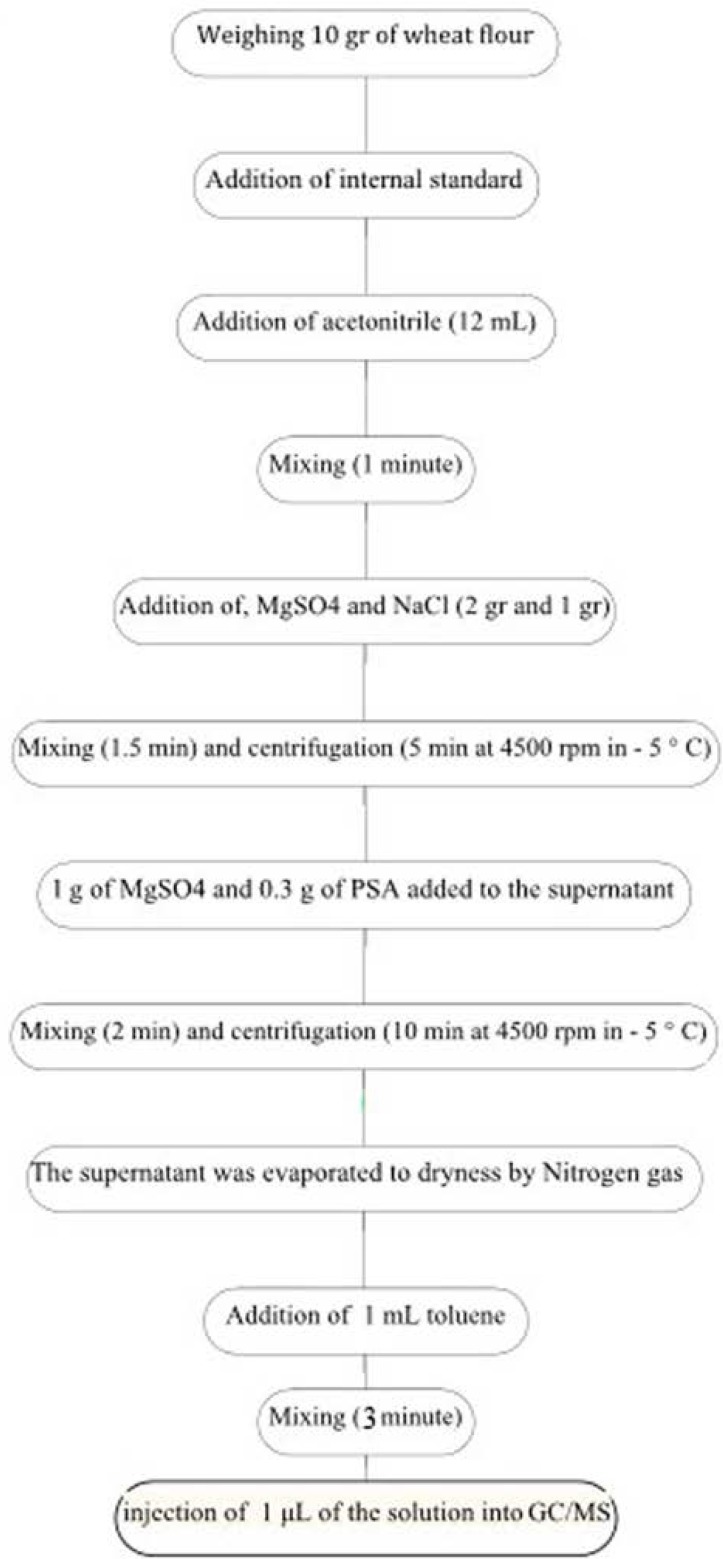
Flow diagram of the procedure of pesticides determination in wheat flour samples by GC/MS method

For sample preparation, an aliquot of 5 μL of internal standard solution (Triphenylmethane: 1000 mg/L) was added to 10 g of wheat flour samples in a warring blender and after being left for 1 h at ambient temperature in dark, 12 mL acetonitrile was added. The mixture was blended at high speed for 1 min. One gram of NaCl and tow grams MgSO4 were added to the mixture and blending was continued for an additional 90 sec. After centrifugation for 5 min at 4500 rpm in – 5 °C, 1 g of Mg SO4 and 0.3 g of PSA added to the supernatant. The mixture was blended at high speed for 2 min. The mixture was centrifuged again. The supernatant was evaporated to dryness by Nitrogen gas. The residue was reconstituted in 1 mL toluene and the mixture was blended at high speed for 3 min, then 2 μL of the solution was injected into gas chromatograph (Figure 1) (18).

**Figure 2:**
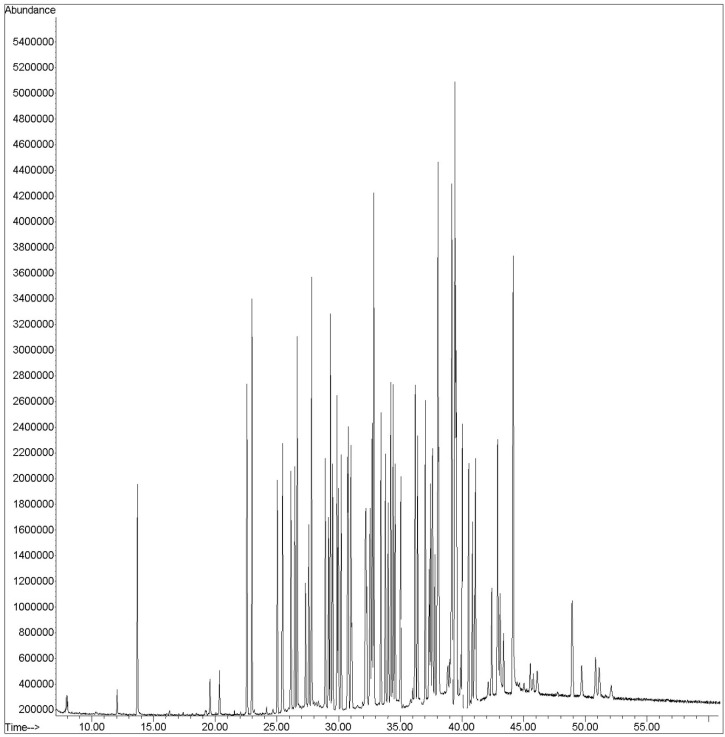
A representative chromatogram obtained for the 58 pesticides and internal standard

**Figure 3:**
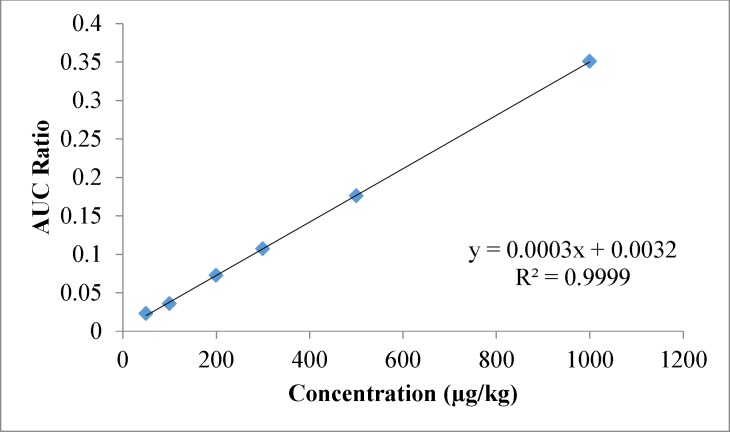
Spiked calibration curve for Propoxure in wheat flour

**Figure 4:**
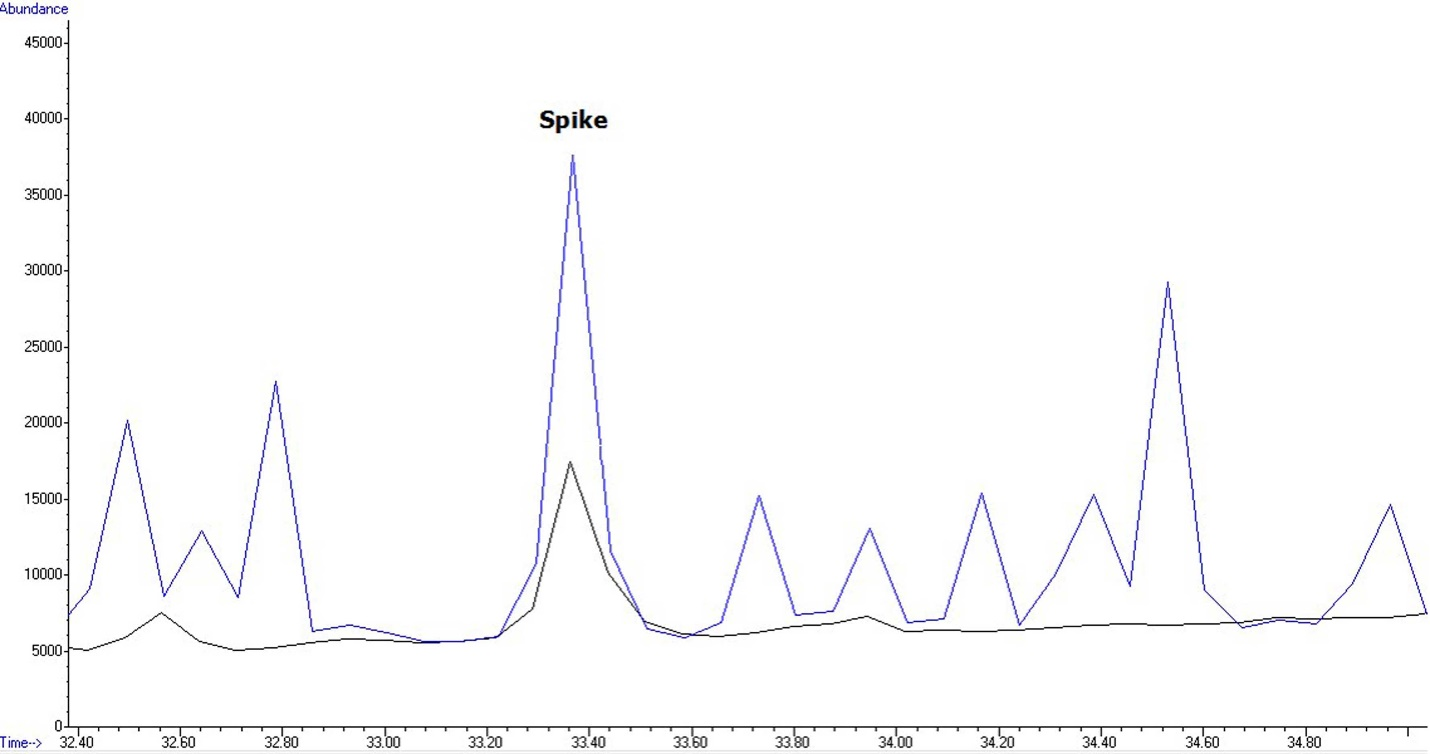
Chromatogram of wheat flour sample spiked with 2, 4 DDE at 200 µg/kg and contaminated baguette flour sample with 2, 4 DDE at 19.88 µg/kg (ppb).

**Figure 5:**
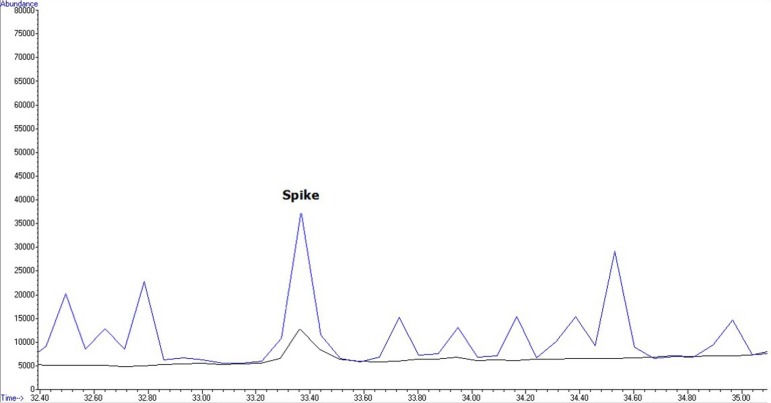
Chromatogram of wheat flour sample spiked with 2, 4 DDE at 200 µg/kg and contaminated baguette flour sample with 2, 4 DDE at 13.7 µg/kg (ppb

**Table 1:** Average recoveries (%) and relative standard deviations (%) obtained by GC-MS analysis of wheat flour at 6 spiking levels (n = 3) in wheat flour samples

**Compound**	**Total average recovery(%), (n=18)**	**Range of RSDr(%) (n=6)**
Propoxure 1	116.60	8.22-15.07
Dichlorvos	99.64	7.13-16.69
Captan	115.77	0.88-19.56
Carbaryl 1	110.06	1.00-18.80
Propoxure 2	108.84	1.20-19.60
Diphenyl amine	110.92	3.80-14.90
Alpha HCH	102.08	5.90-16.60
Dimetoate	99.17	1.72-14.93
Beta HCH	96.10	3.70-14.70
Gamma HCH	102.43	7.40-18.40
Diazinon	114.56	2.00-19.63
Etrimfos	111.56	5.11-18.91
Chlortalonil	101.48	4.92-19.30
Pirimicarb	101.62	3.48-16.21
Chlorpyrifos- methyl	112.17	1.58-12.03
Carbaryl 2	89.68	7.34-19.75
Metalaxyl	104.03	4.20-18.23
Heptachlor	95.34	5.20-19.55
Primiphos methyl	107.82	3.85-13.60
Fenitrothion	106.22	2.80-19.00
Malathion	109.13	3.30-17.80
Fention	109.85	3.40-19.75
Chlorpyrifos	104.28	2.80-18.85
Aldrine	106.33	3.96-19.13
Dicofol	113.57	4.85-16.42
Bioalthrin	100.89	6.16-19.30
Heptachlor-exo-epoxide	109.00	5.69-15.82
Triadimenol 1 and 2	100.01	4.50-13.90
Heptachlor-endo-epoxide	88.48	8.15-19.51
Procymidone	89.42	2.63-17.51
2,4 DDE	101.35	9.09-17.60
Fenamiphos	105.40	5.85-17.25
Alpha-endosulfan	97.25	5.67-17.40
Hexaconazole	106.81	2.34-12.26
4,4 DDE	107.61	6.32-12.56
Dieldrin	100.43	4.89-19.58
Ethion	86.61	5.32-18.15
Trifloxystrobin	102.19	3.51-16.85
Propiconazole 1	91.91	8.08-18.88
Propiconazole 2	106.21	7.71-13.95
Edifenphos	88.80	4.52-14.40
Endosulfan-suiphat	110.65	1.64-18.61
Propargite	115.80	10.49-19.93
Piperonyl butoxide	102.08	2.63-17.10
Tebuconazole	102.48	2.98-18.96
Fenpropathrin	94.73	10.97-18.38
Etoxazole	115.73	7.47-11.79
Fenazaquin	108.26	11.19-18.62
Tetradifon	111.46	4.19-18.49
Phosalon	111.43	2.84-16.91
Azinphos-Methyl	82.46	5.68-17.91
Lambda cyhalothrin	101.46	5.08-14.62
Azinphos- Ethyl	108.30	5.89-18.14
Permethrin 1	104.54	2.80-11.10
Permethrin 2	104.97	1.27-19.96
Fenvalerate 1	102.04	2.43-17.14
Fenvalerate 2	94.43	2.45-18.80
Range	81.46-116.06	0.88-19.96

**Table 2:** Levels of pesticide residues in flour samples collected from the Tehran, Iran, 2014

**Sample**	**Pesticides**	**Concentration(µg/Kg)**	**MRL(µg/Kg)**
Baguette	2,4-DDE	19.88±15.24	50
Barbari	2,4-DDE	13.7±15.24	50
Sangak1	Malathion	50.96±11.38	500
Sangak2	Malathion	62.088±11.38	500

**Table 3:** ADI and EDI of pesticides residue for an adult(µg/kg bw/day).

**Pesticides**	**ADI**	**EDI** a ^،^ c ^،^ d	**ADI%**	**EDI** b ^،^ c ^،^ d	**ADI%**
Malathion	300	0.08	0.02	0.50	0.16
2،4، DDE	-	0.10	-	0.16	-

a The EDI based on the average concentration of contaminants

b The EDI based on maximum contamination

c Calculated on basis of the adult body weight (60 kg)

d Per capita wheat consumption is 482.2 gr/day.

*Chemicals and reagents*

All pesticide standards were purchased from Dr. Ehrenstorfer Co. (Augsburg, Germany). All organic solvents, intended for extraction, were at least of Chromatography grade and purchased from Merck (Darmstadt, Germany). Bulk quantities of anhydrous MgSO4 and NaCl were obtained from Merck (Darmstadt, Germany).

*GC–SQ/MS*

An Agilent Technologies 6890N Network GC System chromatographs (Wilmington, USA) with a SQ mass detector and equipped with an Agilent 7683B autosampler (Agilent technologies, USA) was used. A HP-5 capillary column (30 m × 0.25 mm I.D., 1 μm film thicknesses) was used for separation

*Quality assurance/Quality control*

For quality assurance of the results method validation was carried out according to ICH criteria and for quality assurance of the tests in each run two quality control samples were tested with along with the real samples.

*Calibration standards*

Individual stock standard solutions (10 µg/mL) were prepared in ethyl acetate and stored in dark at – 20 °C. They were kept for 1 h at ambient temperature prior to their use. 

Spiked calibration standards were prepared by addition of 10 μL, 25 μL, 50 μL, 100 μL, 200 μL, 300 μL, 500 μL and 1000 μL of mixed standard stock solution respectively to 10 g of blank wheat flour samples in each case. A stock solution of triphenylmethane (TPM) in ethyl acetate at concentration of 500 µg/L was used as internal standard. An aliquot of 5 μL of TPM solution in ethyl acetate was added to the spiked flour sample as internal standard. The samples so obtained were treated as described in sample preparation section. 

*Recovery studies*

For recovery determination, spiked wheat flour blank samples at concentration levels of 15, 25, 75, 150, 250, 450 and 750 µg/mL were prepared in triplicates and then treated according to the procedure described in sample preparation. Calibration curve was drawn for each pesticide by Excel program 

*GC-SQ–MS analysis*

The GC-SQ-MS was employed with helium as the carrier gas at a constant flow rate of 1 mL/min. The oven temperature started at 75 °C and remained at this temperature for 3 min increasing to 120 °C in a ramp rate of 25 °C/min and then increased to 300 °C at 5 °C/min ramp, holding at 300 °C for 11 min. The injection port was adjusted at 250 °C and the splitless mode was used.

After acquisition of the total ion chromatogram for the mixed stock standard solutions in scan mode, peaks were identified by their retention time and mass spectra. The identification was confirmed by comparing the relative abundances for three ions (one quantifier and two qualifiers) of the experimental standards to the known relative abundances of the Pest Library reference spectra. The most abundant ion that showed no evidence of chromatographic interference and had the highest signal-to-noise ratio was taken for quantification purposes.

*Quantitation*

The concentrations of pesticides were determined by interpolation of the relative peak areas for each pesticide to internal standard peak area in the sample on the spiked calibration curve. In order to compensate for losses during sample processing and instrumental analysis, internal standard (TPM) was used.

*Uncertainty management*

Uncertainty using the coverage factor of 2 and at the confidence level of 95% was expressed (19, 20). 

## Results and Discussion

*Gas chromatographic determination*

Analysis was performed in the SIM mode based on the use of one target and two qualifier ions. Pesticides were identified according to their retention times, target, and qualifier ions. The quantitation was based on the peak area ratio of the targets to that of internal standard. Calibration curves were constructed for each compound using six different concentration levels. For identification of pesticides, the retention time and three ions (one for quantitation and two for identification) were used. A GC–SQ–MS chromatogram of 58 pesticides and internal standard (TPM) analyzed in spiked flour is shown in Figure 2

*Method validation*

*Linearity of the standards in spiked calibration curves*

The 58 pesticides showed linearity in the SIM mode. Linear spiked calibration curves for all the pesticides were obtained with correlation coefficients between 0.977 to 0.999. The spiked calibration curve for Propoxure in wheat flour sample is shown in Figure 3 as a representative

*Limitation of quantification*

Limits of quantification (LOQs) of the proposed method (21) were calculated by considering a value 10 times more than that of background noise in spiked samples at lowest levels. LOQ is the lowest concentration of analyte in a spike sample, which can be quantitatively determined with suitable precision and accuracy.

The limits of detection and limit of quantifications for all the pesticides were < 10 ng/g and < 25 ng/g, respectively. The LOQs for 37 pesticides were 15 ng/g and for the other 21 pesticides were calculated as 25 ng/g in this method.

*Recovery*

Table 1 presents the recovery and repeatability for the six concentration levels. The recovery of pesticides at these six levels was in the range of 81.61-118.41%. In terms of repeatability, the vast majority of pesticides gave RSD < 20% with n = 3 at each spiking level. The recoveries and repeat abilities are in accordance with the criteria set by SANTE Guideline (22)

*Real samples*

For application of the method to real samples, 40 wheat flour samples were purchased from local markets across Tehran in January 2014. Samples were analyzed according to the method described above. For evaluation and analysis, one QC sample at 200 µg/mL was used in each working round. Four of the 40 samples showed contamination with Malathion (2 samples: 50.96 ± 11.38 and 62.088 ± 11.38 ppb) and 2, 4-DDE (2 samples: 19.88±15.24 and 13.7 ± 15.24 ppb), that had levels below MRLs of these pesticides in Iran. Levels of pesticide residue in flour samples collected from Tehran, Iran, 2014 are shown in Table 2 Figures 4 and 5 show an overlaid chromatogram of a spiked flour sample at 200 µg/mL levels and contaminated flour samples in SIM mode.

According to percentages of residues observed in the wheat flour samples, it was determined that 5% of the samples contained 2, 4-DDE and 5% malathion, while the remaining 90% did not contain any pesticide residue. In the present study, residue of malathion and 2, 4-DDE was observed in 4 samples, but concentrations were not higher than the EU MRLs.

Estimated daily intake (EDI) of the determined pesticides residue in wheat was calculated, and compared with the ADI. The estimated daily intake (EDI) of pesticides was determined based on capita consumption of wheat (482.2 g per day) of an adult (body weight taken as 60 kg) as follows: 0.08 for Malathion and 0.1 µg/kg bw/day for 2, 4-DDE (Table 3). The recommended ADI for malathion for an adult is reported to be 300 µg/kg bw/day (23).

The main reason of low pesticide residues in the wheat flour samples in this study, are likely to impact processes such as grinding and debranning of the wheat.

Although the declared value in our study is lower than the recommended ADI, these results do not preclude the need for continuing comprehensive studies for pesticides residue. 

The health risk from consumption of organophosphorus pesticide residue has also been investigated in cereals in other countries. For example, Maver *et al.,* (2007) analyzed organophosphorus pesticide residue of many commodities, including cereals in Slovenia (24). In Another report, Bai *et al.,* (2006) investigated organophosphorus pesticide residue in market foods including cereals in China. They also, found that organophosphorus residue levels were below the MRLs in cereals (25). Bakore *et al*., (2004) reported that all wheat samples were contaminated with various organochlorine pesticide residue in tests conducted in India. The report also stated that aldrin and heptachlor levels were higher than those of the practical limit prescribed by the WHO (26).

Malekirad *et al.,* (2013) reported that farmers who work with organophosphorus pesticide are prone to neuropsychological disorders and diabetes (27).

Taghavian *et al*., (2016) showed that the chronic exposure to organophosphate pesticides can affect the quality of life, depression, anxiety and stress and may endanger their mental health (28).

In the study reported by Toteja *et al*. (2006), residues of DDT (p, p-DDT, o, p-DDT, p, p-DDE) were detected in 78.2% of 632 wheat flour samples but only 1.7% of the total samples analyzed were found to be contaminated with DDT above the maximum residue limit recommended by the Codex (29). In our study, 2,4-DDE was observed in 5% of 40 wheat flour samples, but none of the levels determined in samples exceeded the EU MRLs. Naik *et al*. (2006) states that endosulfan residue in wheat was reported in all samples in India but levels of pesticide residue were well below the MRLs. These results were unlike those of the present study, in which none of the samples were contaminated with endosulfan. A study-conducted by Naik stated that wheat and rice samples taken from a KGS organic farm showed traces of organochlorine pesticide (30). Saeed *et al*., (2001) reported the presence of chlorpyrifos-methyl in most samples containing wheat or wheat flour. Levels in wheat flour (both white and brown) ranged from 37 to 720 ng/g but imported wheat flour was not found to contain any detectable residue. In their study, non-wheat cereals and products did not contain any detectable residue of chlorinated pesticide (31). In our study, none of the wheat flour samples were found to be contaminated with chlorpyrifos-methyl. Guler *et al*. (2010) reported that all the tested wheat samples were contaminated by organochlorine pesticide residue. In some samples levels of organochlorine pesticide residue were higher than limits set by the EU (32). Reports on recent national monitoring programs of pesticide residue in food tested in Europe, USA and Canada, of which data in the US and Canada included imported samples, were all found to have residue in fruit and vegetable products as well as in wheat samples (33-36). Malting is a process applied to cereal grains; it is a combination of two processes; germination and kiln-drying. The condition of pesticides was determined in the malting process from barley to malt. Kaushik *et al.* reported that concentrations of residue after malting ranged from 13–51% for fenitrothion and nuarimol (37). Navarro *et al*. stated that stages of the malting process such as steeping, germination, and kilning contributed to loss of pesticide residue. Steeping was the most important stage in terms of removal of residue (52%) followed by germination (25%) and then kilning (drying and curing) 23% (38). Holland *et al*. (1994), reported that most residue loss occurred during malting on account of high dilution with water (39). 

The bread making process involves two major steps; firstly, yeast-mediated fermentation and then subjection of the substrate to a high temperature for final baking. Both of these factors contribute to degradation of pesticide residue (40). Kaushik *et al*., (2009), observed that in general, the range of pesticide degradation during bread making was highest (75–89%) in samples fortified with 1 ppm. However, variation in residue dissipation of individual pesticides during bread making was observed. At the level of 4 ppm fortification, degradation was in the following order; endosulfan (70%), deltamethrin (63%), malathion (60%), propiaconazole (52%), chlorpyriphos (51%) and hexaconazole (46%), (37).

A weakness of this study was that only 40 samples were included in the study, this constraint was determined by budget. However, the study did include samples from all the major wheat flour sources consumed in Tehran, Iran. 

## Conclusions

In conclusion, the wheat flour consumed in Tehran, was analyzed for 58 pesticides residues by using GC-MS-SIM technique and spike calibration curve. In total 4 samples contained detectable residues (frequency 10%) of pesticides. The residue levels encountered were relatively low and none of these residues exceeded the EU and ISIRI maximum residue limit (MRLs). 

Although, concentration of pesticides in all of the samples under study meets EU limits, still it is recommended to improve residue control at production, tighter regulation of pesticide spraying and also tighter regulation in the sale of pesticides as well as for education of farm personnel and the implementation of integrated pest management methods as ways to reduce contamination with pesticides residue. In this regard, a food control system (i.e. GAP and HACCP). training courses for farmers can be helpful. Moreover, pesticides residue put emphasis on implementing regular monitoring and stricter food safety management system (FSMS). 

The proposed method not only allowed the simultaneous determination and confirmation of 58 pesticides with good recoveries and low detection limits, but also showed to be useful in routine analysis due to its fast and easy procedure. Also, in this study EDI of pesticides in wheat flour was compared with the ADI.

## Competing interests

The authors declare that they have no competing interests.
